# Phase-Matching Quantum Key Distribution with Discrete Phase Randomization

**DOI:** 10.3390/e23050508

**Published:** 2021-04-23

**Authors:** Xiaoxu Zhang, Yang Wang, Musheng Jiang, Yifei Lu, Hongwei Li, Chun Zhou, Wansu Bao

**Affiliations:** 1 Henan Key Laboratory of Quantum Information and Cryptography, SSF IEU, Zhengzhou 450001, China; zxx@qiclab.cn (X.Z.); jms@qiclab.cn (M.J.); lyf@qiclab.cn (Y.L.); lhw@qiclab.cn (H.L.); zc@qiclab.cn (C.Z.); bws@qiclab.cn (W.B.); 2 Synergetic Innovation Center of Quantum Information and Quantum Physics, University of Science and Technology of China, Hefei 230026, China; 3 Basic Department, SSF IEU, Zhengzhou 450001, China

**Keywords:** twin-field quantum key distribution, phase-matching, discrete phase randomization, intrinsic bit error rate

## Abstract

The twin-field quantum key distribution (TF-QKD) protocol and its variations have been proposed to overcome the linear Pirandola–Laurenza–Ottaviani–Banchi (PLOB) bound. One variation called phase-matching QKD (PM-QKD) protocol employs discrete phase randomization and the phase post-compensation technique to improve the key rate quadratically. However, the discrete phase randomization opens a loophole to threaten the actual security. In this paper, we first introduce the unambiguous state discrimination (USD) measurement and the photon-number-splitting (PNS) attack against PM-QKD with imperfect phase randomization. Then, we prove the rigorous security of decoy state PM-QKD with discrete phase randomization. Simulation results show that, considering the intrinsic bit error rate and sifting factor, there is an optimal discrete phase randomization value to guarantee security and performance. Furthermore, as the number of discrete phase randomization increases, the key rate of adopting vacuum and one decoy state approaches infinite decoy states, the key rate between discrete phase randomization and continuous phase randomization is almost the same.

## 1. Introduction

Quantum key distribution (QKD) can offer information theoretically secure means to distribute secret keys between two remote parties [1], but the performance is restricted by the fundamental rate-loss limit [2,3]. Recently, a novel twin-field QKD (TF-QKD) protocol [4] is proposed to surpass the linear Pirandola–Laurenza–Ottaviani–Banchi (PLOB) bound [2], which shows the superiority relation between key rate and channel transmittance, $R∼O(\sqrt{\eta })$. However, the security proof is not completed in the original TF-QKD protocol [4]. In order to present a more rigorous security proof, various variations [5,6,7,8,9,10] of the original TF-QKD protocol have been proposed. The related experimental works have also been extensively studied [11,12,13,14,15,16,17,18,19,20].

All of these variant TF-QKD protocols have their own advantages. The no-phase-post-selection TF-QKD (NPP-TF-QKD) protocol [5,6] provides better key rate performance in closer-to-mid distance, but it needs phase locking and pre-phase feedback in the experiment, so it is hard to implement [5,6,21]. The sending-or-not-sending TF-QKD (SNS-TF-QKD) protocol [10] can tolerate large misalignment errors and provide better performance in long distance [10,21]. The phase-matching QKD (PM-QKD) protocol [8] has no phase locking with phase slices and employs a phase post-compensation technique, so it can be easily experimentally implemented without pre-phase feedback [13,21].

In reality, the decoy state method is adopted to ensure the security of imperfect single photon source [22,23,24,25] in the actual QKD system. An important theoretical premise and assumption of the method is that the global phase of coherent sources should be continuously randomized [26,27,28]. However, perfect phase randomization is very difficult to achieve. In an actual experiment, there are two means to randomize the global phase. One means is to turn the laser on and off by controlling the current, but it is not suitable for PM-QKD with the phase post-compensation technique—the reason for this is that we do not know the precise phase slices. Moreover, experiments show that residual phase correlations may exist between adjacent pulses [29]. The other one is to actively modulate the phase of coherent sources controlled by a phase modulator with a true random number generator; this method is suitable for PM-QKD, but the phase randomization is not continuous. Thus, neither of these two means satisfy the assumption of the decoy state method, which may introduce a potential loophole that threatens the security of the actual protocol [30]. Then, the unambiguous state discrimination (USD) measurement [31] and the photon-number-splitting (PNS) attack [32] can be used against the imperfect phase randomization.

An earlier security analysis of discrete phase randomization appears in the decoy state Bennet-Brassard-1984 (BB84) in Reference [33], which points out, when the number of discrete phase values is larger, that the performance of discrete phase randomization is close to that of continuous phase randomization, and the number is said to be ten [33]. Similar security analysis methods are used for several other protocols, the measurement-device-independent (MDI) QKD in Reference [34], the NPP-TF-QKD in References [35,36], the SNS-TF-QKD in Reference [37], the PM-QKD in Reference [38]. Therein, Reference [38] uses a different security poof method with Reference [8], and there is no in-depth formula derivation in the decoy state PM-QKD with discrete phase randomization. In this paper, we focus on these discrete global phase randomization issues in the PM-QKD protocol [39], study a concrete attack against PM-QKD with imperfect phase randomization, apply the decoy-state method to derive the single photon yield formula to exhibit performance of the key rate and compare the yield difference of continuous phase randomization with discrete phase randomization.

The paper is arranged as follows: in Section 2, we review the PM-QKD protocol in detail, based on the security analysis of symmetric-encoding PM-QKD, we estimate the overall phase error rate. In Section 3, we show a concrete attack against PM-QKD with imperfect phase randomization. In Section 4, we show how to apply the decoy-state method to obtain the upper bound of the phase-flip error rate with discrete phase randomization; moreover, the yield difference between continuous and discrete phase randomization is also studied in this section. The numerical simulation results are shown in Section 5, and then we conclude in Section 6.

## 2. The Protocol of PM-QKD

We employ the attenuated laser as a single photon source, which is regarded as the coherent state. When the coherent state is randomized by continuous phase, it is equivalent to the Fock state, with the photon number distribution as

(1) $$P_{j|\alpha }=e^{-\alpha }\frac{\alpha^{j}}{j!}$$

In this section, we review the PM-QKD protocol, and without considering the security effects of discrete phase randomization, Equation (1) is used for formula derivation.

### 2.1. Protocol Description

The implementation process of the PM-QKD is similar to Reference [39].

- State preparation. In each round, the coherent state $|\sqrt{\alpha_{A}}e^{i(\pi \kappa_{A}+\frac{2\pi }{D}d_{A})}\rangle$ is prepared by Alice, the intensity $\alpha_{A}\in \left\{\mu_{A},\nu_{A},\omega_{A}\right\}$, where $\mu_{A}$ is she signal state, $\nu_{A}$ is the decoy state, $\omega_{A}$ is the vacuum state, the random key bit $\kappa_{A}\in \left\{0,1\right\}$, the discrete phase randomization number $d_{A}$ is randomly chosen from $\left\{0,1,\cdots ,D-1\right\}$, *D* is the number of maximum discrete phase that is modulated by Alice, for simplicity, assume *D* is an even number. Similarly, Bob prepares the coherent state $|\sqrt{\alpha_{B}}e^{i(\pi \kappa_{B}+\frac{2\pi }{D}d_{B})}\rangle$, therein, $\alpha_{A}=\alpha_{B}=\frac{\alpha }{2}\in \left\{\frac{\mu }{2},\frac{\nu }{2},\frac{\omega }{2}\right\}$.
- Measurement. Alice and Bob send their quantum states to Charlie with transmittances $\eta_{A}$ and $\eta_{B}$, Charlie performs an interference measurement with a beam splitter and records which detector (L or R) clicks.
- Announcement. The detection result is announced by Charlie for each round; the intensity settings $\alpha_{A}$, $\alpha_{B}$ and phase numbers $d_{A}$, $d_{B}$ are also announced by Alice and Bob.
- Sifting. After that, the phase post-compensation method is used by Charlie to calculate and then Charlie announces the phase match pairs. Assume the phase compensation $d_{\delta }\in \left\{0,1,\cdots D/2-1\right\}$, only one of the two detectors clicks is the successful detection. If the left detector clicks and $\left|d_{A}-d_{B}-d_{\delta }\right|\;\mathrm{mod}\;D=0$, Alice and Bob keep $\kappa_{A}$ and $\kappa_{B}$ as the raw key. If the right detector clicks and $\left|d_{A}-d_{B}-d_{\delta }\right|\;\mathrm{mod}\;D=D/2$, Bob flips his key bit $\kappa_{B}$. If $\left|d_{A}-d_{B}-d_{\delta }\right|\;\mathrm{mod}\;D\neq 0,\;D/2$, for simplicity, we discard the phase mismatch pairs.
- Parameter estimation. Alice and Bob estimate the information leakage from the raw data that they have kept.
- Key generation. After reconciling the corresponding key string to perform error correction, Alice and Bob use privacy amplification to produce the final keys.

### 2.2. Phase Error Estimation

The security analysis of asymptotic case is considered, so there are no statistical fluctuations. The analysis method of the phase error rate that we use comes from [39], which is an important new viewpoint of QKD security, establishing the relationship between the symmetric encoding and privacy with the standard phase-error-correction approach [40], and we summarize briefly as follows.

If the joint state $\rho_{\mathrm{AB}}$ is a pure of even or odd state, the symmetric encoding PM-QKD protocol is perfectly private, the phase error rate $E_{ph}=0$, if the joint state $\rho_{\mathrm{AB}}$ is a mixture of even and odd state, $\rho_{\mathrm{AB}}=P_{odd}\rho_{odd}+P_{even}\rho_{even}$, the phase error rate $E_{ph}\neq 0$, the effective detection ratios of odd and even components of signal state are estimated by [39]

(2) $$\begin{array}{c} q_{odd|\mu }=P_{odd|\mu }\frac{Y_{odd|\mu }}{Q_{\mu }}\\ q_{even|\mu }=P_{even|\mu }\frac{Y_{even|\mu }}{Q_{\mu }} \end{array}$$

where $Q_{\mu }=P_{odd|\mu }Y_{odd|\mu }+P_{even|\mu }Y_{even|\mu }$ is the total gain of mixture signal state $\rho_{\mathrm{AB}}$. $Y_{odd|\mu }$ and $Y_{even|\mu }$ are the yield of odd signal state $\rho_{odd}$ and even signal state $\rho_{even}$, respectively. $P_{odd|\mu }$ and $P_{odd|\mu }$ are the signal state probability of odd and even photon numbers.

The overall phase error rate comes from the even components, which is estimated by [39]

(3) $$E_{ph}=P_{even|\mu }\frac{Y_{even|\mu }}{Q_{\mu }}$$

where $P_{even|\mu }$ is given by the above section, $Q_{\mu }$ is given by the experiment results, the important task is to estimate the parameter $Y_{even|\mu }$.

For simplicity, we use phase match pairs and discard phase mismatch pairs, so the upper bound of phase error rate comes from the signal state bounded by

(4) $$E_{ph}\leq 1-q_{1|\mu }$$

where $q_{1|\mu }=P_{1|\mu }\frac{Y_{1|\mu }}{Q_{\mu }}$.

According to the above discussion, we get the final secure key rate by

(5) $$R_{f}=\frac{2}{D}Q_{\mu }\left[1-H_{2}\left(E_{ph}\right)-fH_{2}\left(E_{\mu }\right)\right]$$

where $Q_{\mu }$ is the total gain of the signal state, $E_{ph}$ is the phase error rate of the signal state, $E_{\mu }$ is the bit error rate of the signal state, *f* is the error correction efficiency, $H_{2}\left(x\right)=-x\log_{2}\left(x\right)-\left(1-x\right)\log_{2}\left(1-x\right)$ is the binary entropy function.

## 3. Attack PM-QKD with Imperfect Phase Randomization

Considering the extreme case that Eve knows, the exact phases of the signal and decoy states without phase randomization, the PM-QKD protocol will have a serious security loophole. Due to the signal state and the decoy state not being orthogonal, Eve can use USD measurement to distinguish the signal state and the decoy state with the probability q<1. The optimal success probability [41] of USD measurement on each side is $q_{opt}=1-e^{-{\left|\sqrt{\mu }-\sqrt{v}\right|}^{2}/4}$, which is obtained by performing positive operator valued measurement. After performing USD measurement, Eve measures the number of photons in the pulse and performs a PNS attack.

For the sake of simplicity, we neglect the dark count and the misalignment error, and only consider the channel loss. Without attacking, the gains of the signal state and decoy state are

(6) $$\begin{array}{c} Q_{\mu }=1-e^{-\eta \mu }\\ Q_{v}=1-e^{-\eta v} \end{array}$$

where η is the channel loss.

Under the PNS attack, the gains of the signal state and decoy state are

(7) $$\begin{array}{c} Q_{\mu }^{\mathrm{attack}}=\sum_{j=1}^{\infty }q_{opt}^{2}Z_{j}^{\mu }e^{-\mu }\frac{\mu^{j}}{j!}\\ Q_{v}^{\mathrm{attack}}=\sum_{j=1}^{\infty }q_{opt}^{2}Z_{j}^{v}e^{-v}\frac{v^{j}}{j!} \end{array}$$

where $Z_{j}^{\mu }$ and $Z_{j}^{v}$ represent the probability that Eve forwards *j* photons to the signal state and the decoy state, with *j* as the sum of the photons on both sides.

The simplified upper key rate under the PNS attack is bounded by

(8) $$R^{u}=R_{\mathrm{PNS}}=\sum_{j=1}^{\infty }q_{opt}^{2}Z_{j}^{\mu }e^{-\mu }\frac{\mu^{j}}{j!}\left[1-H_{2}\left(E_{ph}\right)\right]$$

The lower key rate of the simplified Equation (5) is bounded by

(9) $$R_{\mathrm{PM}}^{l}=R_{\mathrm{PM}}=Q_{\mu }\left[1-H_{2}\left(E_{ph}\right)\right]$$

Combining the USD measurement with PNS attack, the security of final key rate without the phase randomized system is vulnerable. We can optimize $Z_{j}^{\mu }$ to let $R_{\mathrm{PM}}^{l}>R^{u}$, especially for long distance communication, due to channel loss is large enough, we can block single photon and release multiple photons. Then, the key rate will be higher than the secure key rate, and information will leak out. Hence, Eve’s goal is to minimize $R^{u}$.

It is worth noting that the attack scheme of USD measurement and PNS attack, which requires the quantum non-demolition measurement [42] about the photon numbers, the lossless channel and the ability of controlling detector efficiency, all of these are beyond the current technology. Ma adopts the beam splitting (BS) attack [43] in Reference [8]. We briefly present his results as follows.

Ma [8] points out, under the BS attack, that the probability of successfully distinguishing the states is $P_{\mathrm{suc}}=1-e^{-(1-\eta )\mu }$. The simplified key rate of PM-QKD is lower bounded by

(10) $$R_{\mathrm{BS}}^{l}=Q_{\mu }e^{-2(1-\eta )\mu }$$

Ma [8] supposes that the photon number channel model exists in PM-QKD, then Gottesman–Lo–Lutkenhaus–Preskill (GLLP) [26] analysis can be used to obtain the formula

(11) $$R_{\mathrm{GLLP}}=Q_{1|\mu }\left[1-H_{2}\left(E_{1|\mu }^{ph}\right)\right]-Q_{\mu }fH_{2}\left(E_{\mu }\right)$$

where $Q_{1|\mu }$ is the gain of the single photon signal state, $E_{1|\mu }^{ph}$ is the phase error rate.

Due to the yield being $Y_{j}=1-{\left(1-\eta \right)}^{j}$, the simplified GLLP key rate is lower bounded by

(12) $$R_{\mathrm{GLLP}}^{l}=R_{\mathrm{GLLP}}=Q_{1|\mu }=\eta \mu e^{-\mu }$$

Final results show that, when η is smaller than a certain value, the GLLP formula cannot hold under the BS attack, so the photon number channel model is invalid. Fortunately, the PM formula can defend against BS attack; the precondition is that the intensity must be weaker.

## 4. The PM-QKD with Discrete Phase Modulation of Coherent State Sources

In this section, we introduce the security analysis of discrete phase randomized PM-QKD. Then, we apply the decoy-state method to derive the single photon yield formula. Finally, we compare the yield difference between continuous phase randomization and discrete phase randomization.

### 4.1. Coherent State with Discrete Phase Randomization

For the coherent state with discrete phase randomization, the joint state of Alice and Bob of PM-QKD is as follows

(13) $${|\psi \rangle }_{\mathrm{AB}}=\sum_{d_{A}=0}^{D-1}{|\sqrt{\alpha_{A}}e^{i(\pi \kappa_{A}+\frac{2\pi }{D}d_{A})}\rangle }_{A}{|\sqrt{\alpha_{B}}e^{i(\pi \kappa_{B}+\frac{2\pi }{D}d_{B})}\rangle }_{B}$$

where $\kappa_{A},\kappa_{B}\in \left\{0,1\right\}$, $\left|d_{A}-d_{B}-d_{\delta }\right|\;\mathrm{mod}\;D=0$ or $\left|d_{A}-d_{B}-d_{\delta }\right|\;\mathrm{mod}\;D=D/2$.

Considering the simple case, $d_{\delta }=0$, then $\left|d_{A}-d_{B}\right|=0$ or $\left|d_{A}-d_{B}\right|=D/2$. Now, the density matrix can be written as

(14) $$\begin{array}{cc} \rho_{\mathrm{AB}}^{D} & =\frac{1}{D}\sum_{d_{A}=0}^{D-1}{|\sqrt{\alpha_{A}}e^{i(\pi \kappa_{A}+\frac{2\pi }{D}d_{A})}\rangle }_{A}\langle \sqrt{\alpha_{A}}e^{-i(\pi \kappa_{A}+\frac{2\pi }{D}d_{A})}|\\ & ⊗{|\sqrt{\alpha_{B}}e^{i(\pi \kappa_{B}+\frac{2\pi }{D}d_{B})}\rangle }_{B}\langle \sqrt{\alpha_{B}}e^{-i(\pi \kappa_{B}+\frac{2\pi }{D}d_{B})}|\\ & =\sum_{j=0}^{D-1}P_{j|\alpha }^{D}{|\lambda_{j|\alpha }^{D}\rangle }_{\mathrm{AB}}\langle \lambda_{j|\alpha }^{D}| \end{array}$$

where $P_{j|\alpha }^{D}=\sum_{l=0}^{\infty }\frac{e^{-\alpha }\alpha^{lD+j}}{(lD+j)!}$, ${|\lambda_{j|\alpha }^{D}\rangle }_{\mathrm{AB}}=\frac{e^{-\alpha /2}}{\sqrt{P_{j|\alpha }^{D}}}\sum_{l=0}^{\infty }\frac{{\left(\sqrt{\alpha }\right)}^{lD+j}}{\sqrt{(lD+j)!}}{|lD+j\rangle }_{\mathrm{AB}}$, with ${|lD+j\rangle }_{\mathrm{AB}}=\frac{1}{\sqrt{2^{lD+j}\left(lD+j\right)}}{\left(a^{†}\pm b^{†}\right)}^{lD+j}{|00\rangle }_{\mathrm{AB}}$.

In our security analysis with discrete phase randomization, we modify the final secure key rate Equation (5) to

(15) $$R_{f}=\frac{2}{D}Q_{\mu }\left[1-H_{2}\left(E_{ph}^{D}\right)-fH_{2}\left(E_{\mu }\right)\right]$$

where the upper bound of phase error rate $E_{ph}^{D}$ comes from the signal state bounded by $E_{ph}^{D}\leq 1-q_{1|\mu }^{D}$, with $q_{1|\mu }^{D}=P_{1|\mu }^{D}\frac{Y_{1|\mu }^{D}}{Q_{\mu }}$. The bit error rate $E_{\mu }$ and the gain $Q_{\mu }$ remain the same.

### 4.2. The Decoy-State Method

In discrete phase randomized PM-QKD, we estimate the yield $Y_{1|\mu }^{D}$ of the single-photon signal state. We use the vacuum and one decoy state, which is similar to the BB84 decoy state analysis [24].

We know that, in the security proof of the decoy state method with continuous phase randomization, there is an important assumption

(16) $$Y_{j|signal}=Y_{j|decoy}$$

However, it is not strict in the condition of discrete phase randomization, $Y_{j|signal}^{D}\neq Y_{j|decoy}^{D}$; the reason lies in

(17) $$|\lambda_{j|\mu }^{D}\rangle \;\neq \;|\lambda_{j|v}^{D}\rangle$$

Consider the properties of trace distance; we need to estimate the difference of yields for different intensities as [33]

(18) $$\left|Y_{j|\mu }^{D}-Y_{j|v}^{D}\right|=\sqrt{1-{\left(F_{j|\mu \nu }^{D}\right)}^{2}}$$

where $F_{j|\mu \nu }^{D}=\sum_{l=0}^{\infty }\frac{{\left(\mu v\right)}^{(lD+j)/2}}{\left(lD+j\right)!}/\sqrt{\sum_{l=0}^{\infty }\frac{\mu^{lD+j}}{\left(lD+j\right)!}\sum_{l=0}^{\infty }\frac{\nu^{lD+j}}{\left(lD+j\right)!}}$, that is the fidelity of $|\lambda_{j|\mu }^{D}\rangle$ and $|\lambda_{j|v}^{D}\rangle$.

The estimation of the yield $Y_{1|\mu }^{D}$ is similar to continuous phase randomization. The equation can be written as

(19) $$\begin{array}{c} Q_{\mu }=\sum_{j=0}^{D-1}P_{j|\mu }^{D}Y_{j|\mu }^{D}\\ Q_{v}=\sum_{j=0}^{D-1}P_{j|v}^{D}Y_{j|v}^{D}=\sum_{j=0}^{N-1}P_{j|v}^{D}Y_{j|\mu }^{D}+\sum_{j=0}^{D-1}P_{j|v}^{D}\left(Y_{j|v}^{D}-Y_{j|\mu }^{D}\right) \end{array}$$

We have

(20) $$\begin{array}{cc} Y_{1|\mu }^{D}= & [P_{2|\mu }^{D}Q_{v}-P_{2|v}^{D}Q_{\mu }-\left(P_{2|\mu }^{D}P_{0|v}^{D}-P_{0|\mu }^{D}P_{2|v}^{D}\right)Y_{0|\mu }^{D}\\ & \;-P_{2|\mu }^{D}\sum_{j=0}^{D-1}P_{j|v}^{D}\left(Y_{j|v}^{D}-Y_{j|\mu }^{D}\right)-\sum_{j\geq 3}^{\infty }\left(P_{2|\mu }^{D}P_{j|v}^{D}-P_{j|\mu }^{D}P_{2|v}^{D}\right)Y_{j|\mu }^{D}]\\ & \;/(P_{2|\mu }^{D}P_{1|v}^{D}-P_{1|\mu }^{D}P_{2|v}^{D}) \end{array}$$

with $\sum_{j\geq 3}^{\infty }\left(P_{2|\mu }^{D}P_{j|v}^{D}-P_{j|\mu }^{D}P_{2|v}^{D}\right)Y_{j|\mu }^{D}\leq 0$, $Y_{0|\mu }^{D}\leq Q_{\omega }/P_{0|\omega }^{D}+\sqrt{1-{\left(F_{0|\mu \omega }^{D}\right)}^{2}}$ and $\sum_{j=0}^{D-1}P_{j|v}^{D}\left(Y_{j|v}^{D}-Y_{j|\mu }^{D}\right)=\sum_{j=0}^{D-1}P_{j|\mu }^{D}\sqrt{1-{F_{j|\mu \nu }^{D}}^{2}}$.

Then

(21) $$Y_{1|\mu }^{D}\geq \frac{P_{2|\mu }^{D}Q_{v}-P_{2|v}^{D}Q_{\mu }-\left(P_{2|\mu }^{D}P_{0|v}^{D}-P_{0|\mu }^{D}P_{2|v}^{D}\right)Y_{0|\mu }^{D}-P_{2|\mu }^{D}\sum_{j=0}^{D-1}P_{j|\mu }^{D}\sqrt{1-{F_{j|\mu \nu }^{D}}^{2}}}{P_{2|\mu }^{D}P_{1|v}^{D}-P_{1|\mu }^{D}P_{2|v}^{D}}$$

### 4.3. The Yield Difference between Continuous and Discrete Phase Randomization

To compare the yield difference of continuous phase randomization and discrete phase randomization, the density matrix of the continuous phase randomization can be written as

(22) $$\begin{array}{cc} \rho_{\mathrm{AB}} & =\frac{1}{2\pi }\int_{0}^{2\pi }{|\sqrt{\alpha_{A}}e^{i(\pi \kappa_{A}+\varphi_{A})}\rangle }_{A}\langle \sqrt{\alpha_{A}}e^{-i(\pi \kappa_{A}+\varphi_{A})}|\\ & ⊗{|\sqrt{\alpha_{B}}e^{i(\pi \kappa_{B}+\varphi_{B})}\rangle }_{B}\langle \sqrt{\alpha_{B}}e^{-i(\pi \kappa_{B}+\varphi_{B})}|\\ & =\sum_{j=0}^{\infty }P_{j|\alpha }{|j\rangle }_{\mathrm{AB}}\langle j| \end{array}$$

where the general Poisson distribution $P_{j|\alpha }$ is given by Equation (1), with ${|j\rangle }_{\mathrm{AB}}=\frac{1}{\sqrt{2^{j}j!}}{\left(a^{†}\pm b^{†}\right)}^{j}{|00\rangle }_{\mathrm{AB}}$.

In the ideal case, D→∞, the fidelity $F_{j|\alpha }^{C,D}$ between ${|j\rangle }_{\mathrm{AB}}$ and ${|\lambda_{j|\alpha }^{D}\rangle }_{\mathrm{AB}}$ should be the same. In the security analysis, the fidelity $F_{j|\alpha }^{C,D}$ between ${|j\rangle }_{\mathrm{AB}}$ and ${|\lambda_{j|\alpha }^{D}\rangle }_{\mathrm{AB}}$ is bounded by

(23) $$\begin{array}{cc} F_{j|\alpha }^{C,D} & =F\left({|j\rangle }_{\mathrm{AB}},{|\lambda_{j|\alpha }^{D}\rangle }_{\mathrm{AB}}\right)=\frac{\left|{\langle j|\lambda_{j|\alpha }^{D}\rangle }_{\mathrm{AB}}\right|}{\sqrt{{\langle j|j\rangle }_{\mathrm{AB}}{\langle \lambda_{j|\alpha }^{D}|\lambda_{j|\alpha }^{D}\rangle }_{\mathrm{AB}}}}\\ & =1/\frac{e^{-\alpha /2}}{\sqrt{P_{j|\alpha }^{D}}}\sum_{l=0}^{\infty }\frac{{\left(\sqrt{\alpha }\right)}^{lD+j}}{\sqrt{(lD+j)!}} \end{array}$$

which is related to the intensity α, photon number *j* and discrete phase numbers *D*.

Therefore, the yield difference is bounded by

(24) $$\left|Y_{j|\alpha }-Y_{j|\alpha }^{D}\right|\leq \sqrt{1-F_{j|\alpha }^{C,D}}=\sqrt{1-1/\frac{e^{-\alpha /2}}{\sqrt{P_{j|\alpha }^{D}}}\sum_{l=0}^{\infty }\frac{{\left(\sqrt{\alpha }\right)}^{lD+j}}{\sqrt{(lD+j)!}}}$$

## 5. Numerical Results

Let’s suppose the transmittances between Alice/Bob and Charlie are $\eta_{A}=\eta_{B}=\eta_{f}$, the detection efficiency of detectors is $\eta_{d}$, after the channel and detection losses, $\eta =\eta_{f}\eta_{d}$, the detection click probabilities are given by

(25) $$\begin{array}{c} P_{\alpha }\left(\bar{L}\right)=\left(1-p_{d}\right)e^{-\eta \alpha \cos^{2}\frac{\phi_{\mathrm{AB}}}{2}}\\ P_{\alpha }\left(L\right)=1-P_{\alpha }\left(\bar{L}\right)\\ P_{\alpha }\left(\bar{R}\right)=\left(1-p_{d}\right)e^{-\eta \alpha \sin^{2}\frac{\phi_{\mathrm{AB}}}{2}}\\ P_{\alpha }\left(R\right)=1-P_{\alpha }\left(\bar{R}\right) \end{array}$$

where $P_{\alpha }\left(L\right)/P_{\alpha }\left(R\right)$ and $P_{\alpha }\left(\bar{L}\right)/P_{\alpha }\left(\bar{R}\right)$ are the detection click probabilities of the L/R click and no L/R click, $\phi_{\mathrm{AB}}$ is the phase mismatch between Alice and Bob.

Due to the discrete phase randomization, we can obtain *D* phase slices. Although we keep the phase match pairs and discard all of the others, there is still an intrinsic bit error rate [4], $E_{D}=\frac{D}{2\pi }\int_{0}^{2\pi /D}\sin^{2}\frac{\phi_{\mathrm{AB}}}{2}d\phi_{\mathrm{AB}}$. Significantly, this is very different from BB84 protocol with the global phase mismatch value $\phi_{\mathrm{AB}}=0$. When we use discrete phase randomization, we must consider the intrinsic bit error rate, which will deeply affect the bit error rate and phase error rate.

The error gain can be given by

(26) $$\begin{array}{c} Q_{\alpha }^{E}=\frac{D}{2\pi }\int_{0}^{\frac{2\pi }{D}}P_{\alpha }\left(R\right)P_{\alpha }\left(\bar{L}\right)d\phi_{\mathrm{AB}}\\ =\frac{D}{2\pi }\int_{0}^{\frac{2\pi }{D}}\left(1-p_{d}\right)e^{-\eta \alpha \cos^{2}\frac{\phi_{\mathrm{AB}}}{2}}d\phi_{\mathrm{AB}}-{\left(1-p_{d}\right)}^{2}e^{-\eta \alpha } \end{array}$$

We can derive the total gain $Q_{\alpha }$ as

(27) $$\begin{array}{c} Q_{\alpha }=\frac{D}{2\pi }\int_{0}^{\frac{2\pi }{D}}[P_{\alpha }\left(L\right)P_{\alpha }\left(\bar{R}\right)+P_{\alpha }\left(R\right)P_{\alpha }\left(\bar{L}\right)]d\phi_{\mathrm{AB}}\\ =\frac{D}{2\pi }\int_{0}^{\frac{2\pi }{D}}\left(1-p_{d}\right)e^{-\eta \alpha \sin^{2}\frac{\phi_{\mathrm{AB}}}{2}}d\phi_{\mathrm{AB}}-{\left(1-p_{d}\right)}^{2}e^{-\eta \alpha }+Q_{\alpha }^{E} \end{array}$$

The bit error rate of signal states is given by

(28) $$E_{\mu }=\frac{Q_{\mu }^{E}\left(1-2e_{opt}\right)+e_{opt}Q_{\mu }}{Q_{\mu }}$$

The simulate parameters are listed in Table 1.

**Figure 1:**
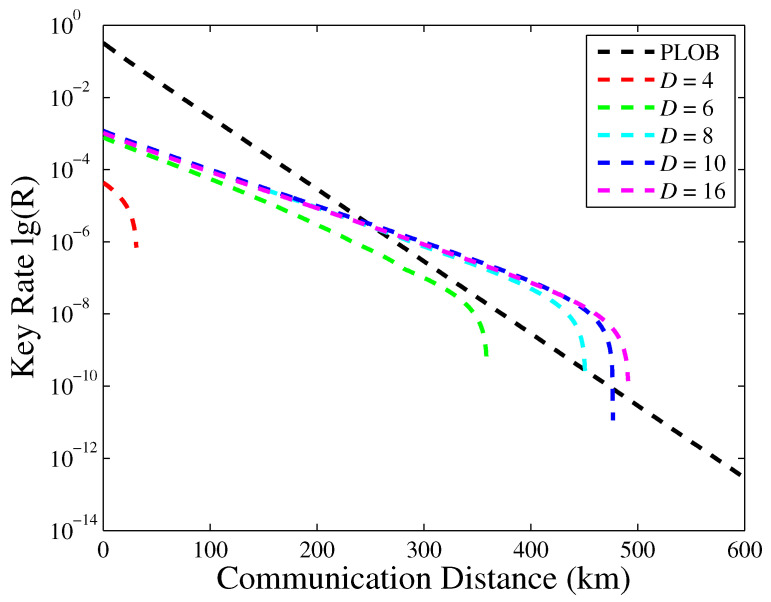
The key rate versus the transmission distance of the PM-QKD with different number of discrete phase values; the PLOB linear bound is plotted for comparison.

In the key rate versus the transmission distance of the finite decoy states PM protocol with a different number of phase values, as shown in Figure 1, the PLOB bound is plotted for comparison. The smaller *D*, the lower the key rate; the reason is that the smaller the *D*, the larger the intrinsic bit error rate. D=8 can break the PLOB bound, and meanwhile, we can find that there is an optimal D=10, which can guarantee better performance. With the increase of *D*, the key rate will become lower due to the sifting factor 2/D. Hence, in an actual experiment of PM-QKD, we must find the suitable discrete phases value to guarantee security and performance. When D→∞, the key rate will tend to 0; we do not present it here.

**Figure 2:**
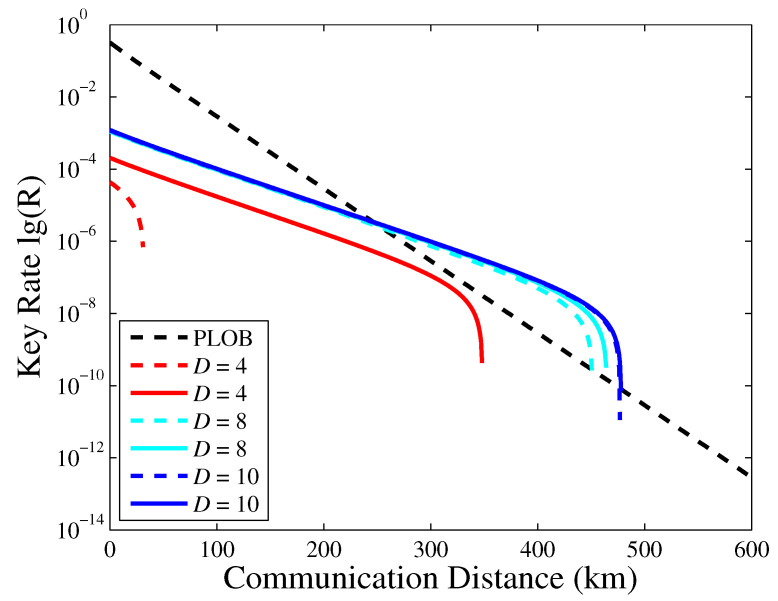
The key rate versus the transmission distance of the PM-QKD with different number of discrete phase values, infinite decoy states and vacuum and one decoy state are plotted for comparison. The dash line represents the case of vacuum and one decoy state; the solid line represents the case of infinite decoy states.

Moreover, we compare the performance of PM-QKD with discrete phase randomization between infinite decoy states and vacuum and one decoy state. As depicted in Figure 2, when we adopt vacuum and one decoy state and small *D*, the key rate exhibits poor performance. As *D* increases, the key rate of adopting vacuum and one decoy state approaches infinite decoy states. Combining the conclusion of Figure 1, we find that the discrete phase D=10 still maintains good security and performance when the finite decoy states are implemented.

**Figure 3:**
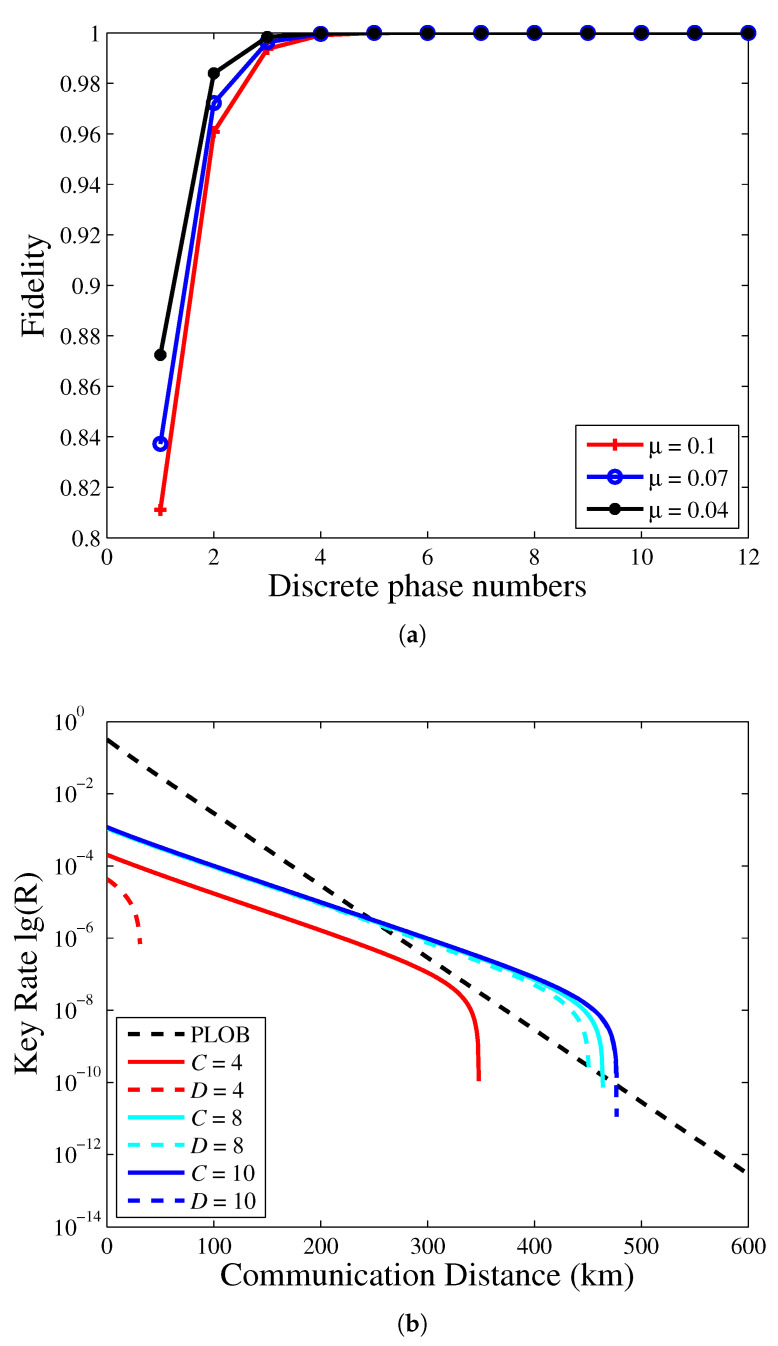
(**a**) The fidelity of different mean photon numbers. The fidelity refers to Equation (23), which we take j=1. (**b**) The key rate versus the transmission distance of the PM-QKD with a different number of discrete phase values. The solid line represents the coherent state with continuous phase randomization; the dash line represents the coherent state with discrete phase randomization.

Due to there being a sifting factor 2/D, we know that when D→∞, the key rate will tend to 0. In order to compare the key rate between continuous phase randomization and discrete phase randomization, we first compare the fidelity between ${|j\rangle }_{\mathrm{AB}}$ and ${|\lambda_{j|\alpha }^{D}\rangle }_{\mathrm{AB}}$, as shown in Figure 3a. The fidelity varies slightly with the intensity. With the increase of *D*, the fidelity gradually approaches 1. Therefore, when *D* is too small, the method of continuous phase randomization is not suitable; we cannot ignore the safety effect of discrete phase randomization.

Then, considering finite decoy states, the key rate between continuous phase randomization and discrete phase randomization has been studied in Figure 3b. As *D* increases, the performance of a key rate between discrete phase randomization and continuous phase randomization is almost the same. This is consistent with the conclusion in Figure 3a.

## 6. Conclusions

In this paper, we introduce the USD measurement and PNS attack against PM-QKD with imperfect phase randomization, and simultaneously, we deeply study the security of discrete phase randomization PM-QKD protocol with a decoy state in the asymptotic case. Our simulation results show that, as *D* increases, the key rate of adopting vacuum and one decoy state approaches infinite decoy states, and furthermore, the performance of key rate between discrete phase randomization and continuous phase randomization is almost the same. We also find that due to the intrinsic bit error rate and sifting factor, there is an optimal discrete phase randomization value to guarantee security and performance. Therefore, for the actual PM-QKD system, we should better adopt the suitable discrete phase randomization value to apply. 

## Figures and Tables

**Table 1:** List of parameters used in numerical simulations. Here $p_{d}$ is the dark counts rate; $e_{opt}$ is the misalignment error probability of the system; $\eta_{d}$ is the detection efficiency; *f* is the error correction efficiency; $\eta_{f}$ is the transmission fiber loss coefficient (dB/km).

$p_{d}$	$e_{opt}$	$\eta_{d}$	*f*	$\eta_{f}$
$1\times 10^{-8}$	1.5%	0.2	1.1	0.2

## Data Availability

The data presented in this study are available within the article.
